# A New Mutation, *hap1-2*, Reveals a C Terminal Domain Function in AtMago Protein and Its Biological Effects in Male Gametophyte Development in *Arabidopsis thaliana*

**DOI:** 10.1371/journal.pone.0148200

**Published:** 2016-02-11

**Authors:** Kevin Cilano, Zachary Mazanek, Mahmuda Khan, Sarah Metcalfe, Xiao-Ning Zhang

**Affiliations:** 1 Department of Biology, Saint Bonaventure University, Saint Bonaventure, New York, United States of America; 2 Biochemistry Program, Saint Bonaventure University, Saint Bonaventure, New York, United States of America; Institute of Botany, Chinese Academy of Sciences, CHINA

## Abstract

The exon-exon junction complex (EJC) is a conserved eukaryotic multiprotein complex that examines the quality of and determines the availability of messenger RNAs (mRNAs) posttranscriptionally. Four proteins, MAGO, Y14, eIF4AIII and BTZ, function as core components of the EJC. The mechanisms of their interactions and the biological indications of these interactions are still poorly understood in plants. A new mutation, *hap1-2*. leads to premature pollen death and a reduced seed production in Arabidopsis. This mutation introduces a viable truncated transcript *AtMagoΔC*. This truncation abolishes the interaction between AtMago and AtY14 *in vitro*, but not the interaction between AtMago and AteIF4AIII. In addition to a strong nuclear presence of AtMago, both AtMago and AtMagoΔC exhibit processing-body (P-body) localization. This indicates that AtMagoΔC may replace AtMago in the EJC when aberrant transcripts are to be degraded. When introducing an NMD mutation, *upf3-1*, into the existing *HAP1/hap1-2* mutant, plants showed a severely reduced fertility. However, the change of splicing pattern of a subset of SR protein transcripts is mostly correlated with the *sr45-1* and *upf3-1* mutations, not the *hap1-2* mutation. These results imply that the C terminal domain (CTD) of AtMago is required for the AtMago-AtY14 heterodimerization during EJC assembly, UPF3-mediated NMD pathway and the AtMago-AtY14 heterodimerization work synergistically to regulate male gametophyte development in plants.

## Introduction

The quality and the viability of mRNAs are vital for translation success in living cells. In plants, the majority of the nuclear genes contain introns [1]. These introns are spliced out by the spliceosome to yield mature mRNAs. The spliceosome then recruits eIF4AIII, a component of exon-exon junction complex (EJC) [2]. This leads to the EJC assembly on the transcript. The EJC is a conserved multiprotein complex in all eukaryotes. Four proteins, Mago, Y14, eIF4AIII and Barentsz (BTZ), form the core of the EJC, while other factors, such as RNPS1, UPF3, TAP/NXF/p15, REF/Aly, SRm160, bind to the core peripherally [3]. It has been shown that the EJC plays a critical role in mRNA quality control and metabolism, such as mRNA splicing, localization, export and nonsense-mediated decay (NMD) [3–6]. The EJC associates with the mRNA molecule in a position-specific manner during splicing, at the conserved position ~20–24 nucleotides upstream of the exon-exon junction, and remains bound until translation [7]. In *Drosophila* and Hela cells, EJC-associated mRNAs are transported from the nucleus to the cytoplasm [4]. Mutations in Mago or Y14 caused a failure in localizing *oskar* mRNA to the posterior of the oocyte during oogenesis and the zygote lethality [8, 9]. Recently, an interesting study reported that the splicing of the *MAPK* mRNA in *Drosophila* required functional Mago [10], hinting that the EJC may favor some targets over others. The EJC orthologs are also found necessary in both plants and animals for intron-based NMD [11, 12]. Studies in the protein structure of *Drosophila* and human EJC core components showed that Mago and Y14 form a stable heterodimer before binding to eIF4AIII and BTZ, while eIF4AIII, a DEAD-box RNA helicase, binds to the RNA molecule in the presence of ATP [13, 14]. Although it seems that the Mago-Y14 heterodimer contributes to the EJC function by preventing ATP hydrolysis [14], it is also possible that all four core proteins work together to keep the mRNA molecule bound regardless of the status of ATP hydrolysis [15]. The exact mechanism for trapping the EJC on the mRNA molecule is still in debate.

Mago was originally characterized in *Drosophila* as a requirement for germ line cell differentiation and embryogenesis in various animal models [16, 17]. In addition to the maternal effect observed in *Drosophila*, Mago was shown to be required for the asymmetric localization of specific mRNAs during spermatogenesis in a water fern species, *Marsilea vestita* [18]. Meanwhile, the Arabidopsis *Mago* (*AtMago*), also known as *HAPLESS1* (*HAP1*), was found to be required for pollen tube guidance [19]. When Mago was knocked down, a systemic abnormality was observed at various developmental stages, particularly in reproduction [20–22]. Although the protein-protein interactions within the EJC have been extensively studied in animal models, the mechanisms by which the EJC assembles and functions in plant cells are still poorly understood.

In angiosperms, haploid cells only exist in the male gametophyte (pollen) and the female gametophyte (embryo sac). During pollen development, pollen mother cells undergo meiosis to yield haploid pollen tetrads. Each microspore from the tetrad divides twice to form a tricellular pollen grain that encloses a vegetative cell and two sperms. The whole process involves intensive signaling and communication among the cells [23]. Since pollen grains are to leave the anther upon maturation, all the mRNA and protein molecules required for the pollen tube germination, guidance and the fertilization must be premade and packaged in the pollen before maturation. Thus, the quality and quantity of these products are crucial for reproductive success in a plant. This makes the haploid pollen an excellent system to study the mechanisms and the functions of the EJC.

In this study, we examined male gametophyte defects associated with a new *AtMago* mutation, *hap1-2*. An effect of this mutation was revealed in the haploid pollen. An aberrant transcript accumulated in the *hap1-2* heterozygous mutant, and pollen development was defective. The possible mechanisms underlining these defects are proposed accordingly.

## Materials and Methods

### Plant Growth Condition

All *Arabidopsis* plants used in this study are in *Colombia* (Col) background. Mutant plants, *qrt;HAP1/hap1-2* (SAIL_269_C02), *sr45-1* (SALK_004132) and *upf3-1* (SALK_025175) were provided by *Arabidopsis Biological Resource Center* (ABRC). Primers for examining T-DNA insertion in each mutant were designed by the T-DNA Primer Design Tool powered by Genome Express Browser Server (S1 Table). Primers used to examine T-DNA insertion in *sr45-1* were as described previously [24]. All plants were grown in soil with 16/8 hr photoperiod at 100 μmol m^-2^ s^-1^. Peter’s fertilizer (Griffin Greenhouse & Nursery Supplies, 67–2030) was applied once at the concentration of 3 g L^-1^ after the first week. All plants were grown at 24°C.

### RNA Isolation and RT-PCR

RNeasy Mini Kit (Qiagen) was used to isolate total RNAs. About five microgram of total RNA from each sample was digested by DNase I (Invitrogen) and applied for reverse transcription with Superscript II system from Invitrogen. The transcription level of *AtMago* and *AtMagoΔC* in different mutants and/or transgenic plants was verified by either semi quantitative PCR on Master Pro ThermoCycler (Eppendorf) or quantitative PCR on Chromo 4 ThermoCycler (Bio-Rad Inc.) with primers AtMagoF and AtMagoR for *AtMago*, and primers AtMagoF and AtMagoΔCR for *AtMagoΔC*. Power SYBR Green PCR Master Mix (Invitrogen) was used to prepare all reactions for quantitative PCR reactions. *GAPDH* was used for normalization purpose. Splicing pattern of SR protein genes was examined using gene specific primers. The expression of *SR45* and *UPF3* was examined with their own specific primers. All primers used are listed in S1 Table.

### 3’ Rapid Amplification of cDNA End (3’ RACE)

Total RNAs were isolated from both *qrt* and *qrt;HAP1/hap1-2* inflorescence tissue using RNeasy Mini Prep kit from Qiagen. 3’ RACE was carried out using 3’ RACE kit from Invitrogen. Primers AtMagoUTRF and AP were used for the first round PCR reaction. Primers AtMagoF and AP were used for the nested PCR reaction. Sequences of the distinct PCR product amplified from *qrt;HAP1/hap1-2* were confirmed at Genewiz Inc.

### Cloning of AtMago, AtMagoΔC, AtY14, AteIF4AIII and DCP1-CFP cDNAs

*AtMago* and *AtMagoΔC* cDNAs were first amplified from *qrt;HAP1/hap1-2* mutant cDNAs using primers *AtMagoFBamHI* and *AtMagoRHindIII* (*AtMago*) or *AtMagoFBamHI* and *AtMagoΔCRHindIII* (*AtMagoΔC*), respectively. The PCR products were cloned into pCRII-TOPO vector (Invitrogen). Then *AtMago* and *AtMagoΔC* were isolated by *Hin*d III and *Bam*H I and cloned into pMAL-c2 expression vector (NEB). *AtY14* and *AteIF4AIII* were amplified from *qrt* cDNAs using *AtY14FNdeI* and *AtY14RSalI* primer pair and *AteIF4AIIIFNdeI* and *AteIF4AIIIRSalI* primer pair, respectively. The PCR products were cloned into pCRII-TOPO vector. Then *AtY14* and *AteIF4AIII* were isolated by *Nde* I and *Sal* I. The released fragments were subsequently cloned into pET16b expression vector (Novagen). *GFP*, AtMago and *AtMagoΔC* were amplified separately, fused together by overlapping PCR to yield *GFP-AtMago* and *GFP-AtMagoΔC*. Then *GFP-AtMago* and *GFP-AtMagoΔC* were cloned between a *35S* promoter and a *NOS* terminator on an expression vector described before [24]. *CFP* and *DCP1* cDNA were also amplified separately, fused together by overlapping PCR to yield *CFP-DCP1* fusion gene. Then *CFP-DCP1* was cloned between a *35S* promoter and a *NOS* terminator as described before [24]. All sequences were confirmed by sequencing at Genewiz Inc. All primer sequences were listed in S1 Table.

### Protein Expression, Purification and in vitro Pull-down

The pMALc2-AtMago and pMALc2-AtMagoΔC constructs were transformed into *E*. *coli* TB1. The pET16b-AtY14 and pET16b-AteIF4AIII constructs were transformed into *E*. *coli* BL21 (DE3). 0.3 μM IPTG was used for protein induction at 25–30°C over night. Then the bacterial cells were harvested, washed in 1xPBS once and resuspended in either Binding Buffer (400 mM NaCl, PBS, 20 mM Imidazole) for the pET16b-AtY14 and pET16b-AteIF4AIII constructs or Column Buffer (200 mM NaCl, 1mM EDTA, 20 mM Tris-HCl, pH7.4) for the pMALc2 constructs. Lysozyme was added to 1 mg/ mL. The lysozyme/bacterial suspensions were let incubate on ice for 30 mins then frozen in -80°C over night. After all the suspensions were thaw the next day, the cell lysate was obtained from each suspension after votex and centrifuge at 10,000 *g* for 20 mins for further protein purification. Bacteria harboring pMALc2 vector was used for negative control. His-AtY14 and AteIF4AIII were purified and eluted with ProBond^™^ beads as per the manufacture’s instruction (Invitrogen). MBP, MBP-AtMago and MBP-AtMagoΔC were purified with Amylose beads as per the manufacture’s instruction and kept bond to the beads (NEB). Equal volume of His-Y14 or His-eIF4AIII was diluted in Column Buffer to yield a total of 300 μL volume and incubated with each protein-amylose beads on ice for 1 hour. Beads were then washed in Column Buffer three times before mixed with 2xSDS loading buffer for SDS-PAGE.

### SDS-PAGE and Western Blot

12% SDS-PAGE was used for protein separation. Proteins were then transferred onto nitrocellulose membrane. Western blot was performed with either rabbit Anti-His (Gene Script Inc., 1:1000) or rabbit Anti-MBP (NEB 1:10,000) as the primary antibody and Anti-rabbit-AP (Molecular Probe, 1:3000) as the secondary antibody. NBT/BCIP was used as substrates for AP detection.

### Plant Transformation Screening, Isolation of Transgenic Plants and Genetic Crosses

The *Agrobacteria harboring HAP1* gene expressing plasmid was described previously [19]. Then the *Agrobacteria* was used to transform *HAP1/hap1-2* plants by flower dipping methods described before [24]. Kanamycin resistance was used for stable transgenic plants selection in T1 generation followed by GUS staining assay in the pollen. Three independent transgenic lines were used for further analysis. GUS staining was carried out in T2 and T3 generation in order to select for plants that are homozygous for *hap1-2* mutation and the transgene. All characterization was done at T4 or later generation. CFP-DCP1, GFP-AtMago and GFP-AtMagoΔC constructs were used to create separate stable transgenic lines. Multiple independent lines were examined for each transgene to determine the expression pattern. A positive DCP1-CFP transgenic line was crossed with GFP-AtMago and GFP-AtMagoΔC transgenic lines, respectively. HTR12-GFP plants were crossed with *qrt;HAP1/hap1-2*. Homozygous F3 plants were isolated and used to examine HTR12-GFP expression pattern.

### Confocal Imaging

Nikon Eclipse T*i* confocal microscope was used for all fluorescence imaging except for DAPI. The software EZ C1 3.91 was used to view and record images. These files were processed with the software NIS-Element AR3.2, and figure panels were assembled in Photoshop.

### Pollen Staining

Pollen grains were collected and incubated in GUS staining solution (0.05 mM PBS, pH7.0) with 1 mM 5-bromo-4-chloro-3-indolyl-β-D-Glucopyranosiduronic acid (X-gluc) at 37°C for 15 mins. Then the stained pollen samples were directly mounted on a glass slide for examination under either a Zeiss Axioskop microscope or a Nikon Eclipse Ti microscope. For DAPI staining, pollen tetrads from varies flower stages were released into DAPI solution on a glass slide and observed with a Zeiss Axioskop fluorescence microscope.

### Homology Modeling

The protein structure of AtMago and AtMagoΔC were generated using SWISS-MODEL 8.05 [25–28]. The PDB-ID of the template was 1OO0.

## Protein Sequence Multi-Alignment

The Mago protein sequence from 9 different species was used for multi-alignment using CLC Sequence Viewer 6. These sequences were obtained from the GenBank/EMBL data libraries under accession numbers: EDP02329.1 (*Chlamydomonas reinhardtii*), AAW78462.1 (*Physcomitrella patens*), AAK15756.1 (*Marsilea vestita*), AEE27388.1 (*Arabidopsis thaliana*), ABA97757.1 (*Oryza sativa*.*1*), BAD09395.1 (*Oryza sativa*.*2*), CAB03239.1 (*Caenorhabditis elegans*), AAF46677.1 (*Drosophila melanogaster*), NP_079840.2 (*Mus musculus*), and NP_060518.1 (*Homo sapiens*).

## Results

### The *hap1-2* mutation causes pollen abortion

**Fig 1:**
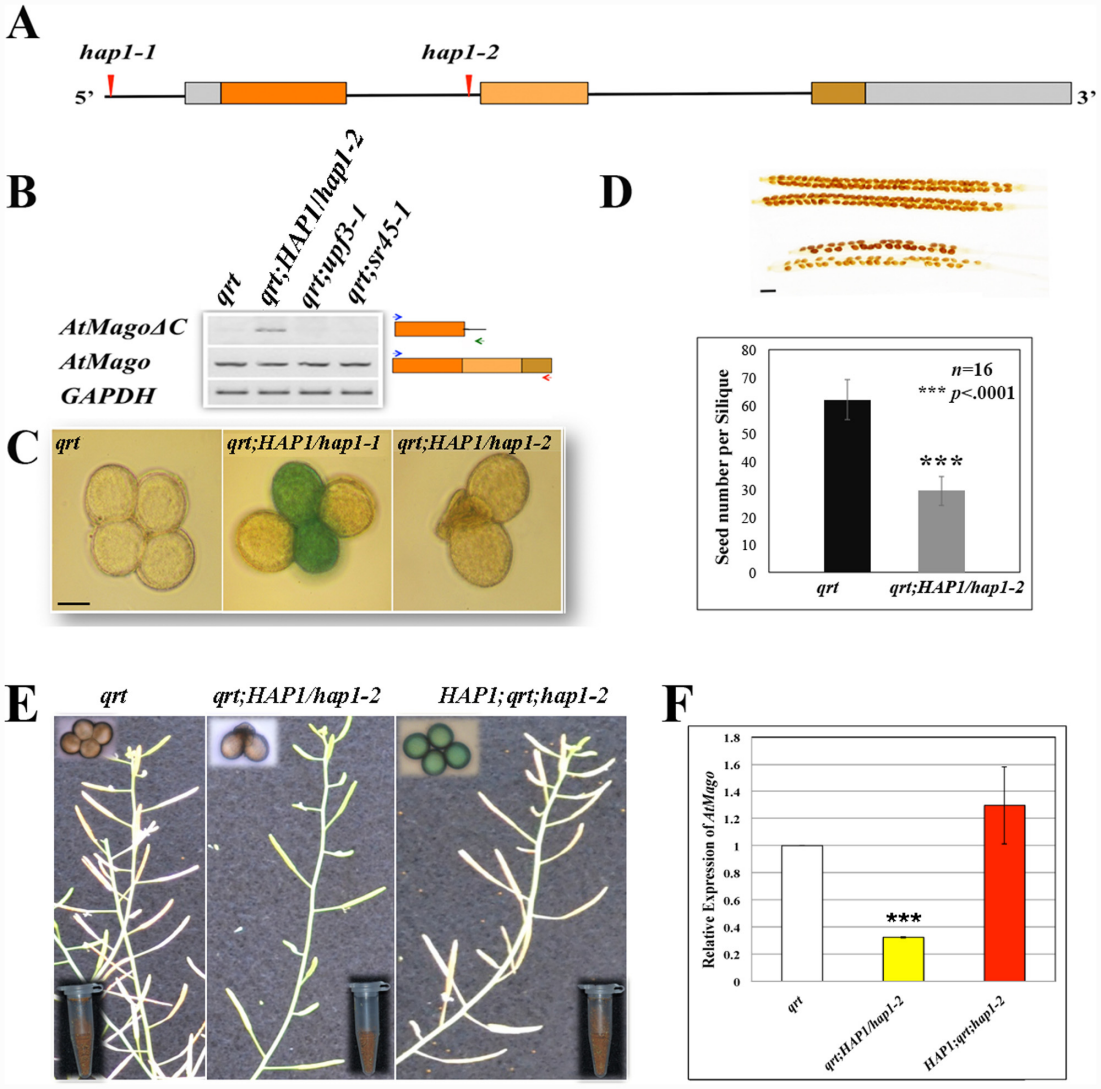
Characterization and complementation of the *HAP1/hap1-2* mutant. (A) *HAP1* gene structure. Exons were shown as colored boxes; introns were shown as straight lines; UTRs were shown as gray boxes. The T-DNA insertion in *hap1-1* and *hap1-2* was indicated by red arrow. (B) rtPCR results showed the expression of a full-length transcript (*AtMago*), a truncated transcript (*AtMagoΔC*) and *GAPDH* in *qrt*, *qrt;HAP1/hap1-2*, *qrt;sr45-1* and *qrt;upf3-1* mutant plants. Total RNAs were extracted from the inflorescence tissue. The exon composition of the transcript and the position of used primers were shown next to the corresponding rtPCR results. **(C)** GUS stained pollen grains from *qrt*, *qrt;HAP1/hap1-1* and *qrt;HAP1/hap1-2* showing the identity of mutant pollen grains. Scale bar = 20 μm. (D) Seed number per silique in *qrt* and *qrt;HAP1/hap1-2* plants. Scale bar = 1 mm. The quantification was done with sixteen siliques. Student t-test was used for statistical analysis. Error bars represent standard deviations. (E) *HAP1* gene complemented *hap1-2* mutant phenotype. The mature plants, GUS-stained pollen grains and seed yield per plant were shown in *qrt*, *qrt;HAP1/hap1-2*, *HAP1;qrt; hap1-2*. (F) qPCR results showing that the *AtMago* gene expression level was fully recovered in the transgenic plant. *GAPDH* was used as control. Three biological replicates were used in the analysis. Error bar showed standard deviation. Statistical significance compared to *qrt* was measured by student t-test (*p* <0.001).

There are three exons and two introns in the *HAP1/AtMago* gene. Compared to the previously described *hap1-1* mutation in the promoter region [19], the *hap1-2* mutant is a result of a T-DNA pCSA110 insertion (Sail_269_C02) 35 nucleotides upstream of the 2^nd^ exon (Fig 1A). The effect of *hap1-2* mutation was visualized in the *quartet* (*qrt*) mutant background because *qrt* releases pollen tetrads upon maturation. pCSA110 contains a pollen-specific reporter construct, *LAT52*::*GUS*, which is turned on during the late stage of pollen development [29]. As described earlier [19], the *hap1-1* mutant pollen was able to develop until maturation and to activate *LAT52*. Strikingly, the *hap1-2* pollen shriveled and ceased to develop before *LAT52* became active (Fig 1C). This leads to a reduced rate of seed formation that is due to the nonexistence of the homozygous mutant. (Fig 1D).

In order to dissect the molecular basis for the pollen lethality in the *hap1-2* mutant pollen, a 3’ RACE assay was carried out to look for alternative transcripts. The search yielded a truncated transcript, *AtMagoΔC*. A premature stop codon was introduced in *AtMagoΔC* by the sequence within the partially retained intron (S1A Fig). Further analysis showed that *AtMagoΔC* was detectable only in plants that carry the *hap1-2* mutation, but not in mutants with defects in splicing (*sr45-1*), or NMD (*upf3-1*) (Fig 1B). However, full length *AtMago* was found in all genotypes examined (Fig 1B). This confirmed that *AtMagoΔC* is indeed a novel transcript instead of a contamination from non-spliced pre-mRNAs. Introducing a copy of the wild type *HAP1* gene successfully recovered the aborted mutant pollen phenotype in the *hap1-2* mutant. The transgenic plants were able to produce viable mutant pollen grains expressing *LAT52*::*GUS* (Fig 1E). These pollen grains were fertile, and the transgenic plants had a similar seed yield to the wild type plants (Fig 1E). The expression of *HAP1* gene was also restored to a comparable level as in the wild type (Fig 1F). This confirmed that the pollen abortion phenotype was indeed due to the *hap1-2* mutation.

### The *hap1-2* pollen fails to sustain normal generative nuclei at early stages

**Fig 2:**
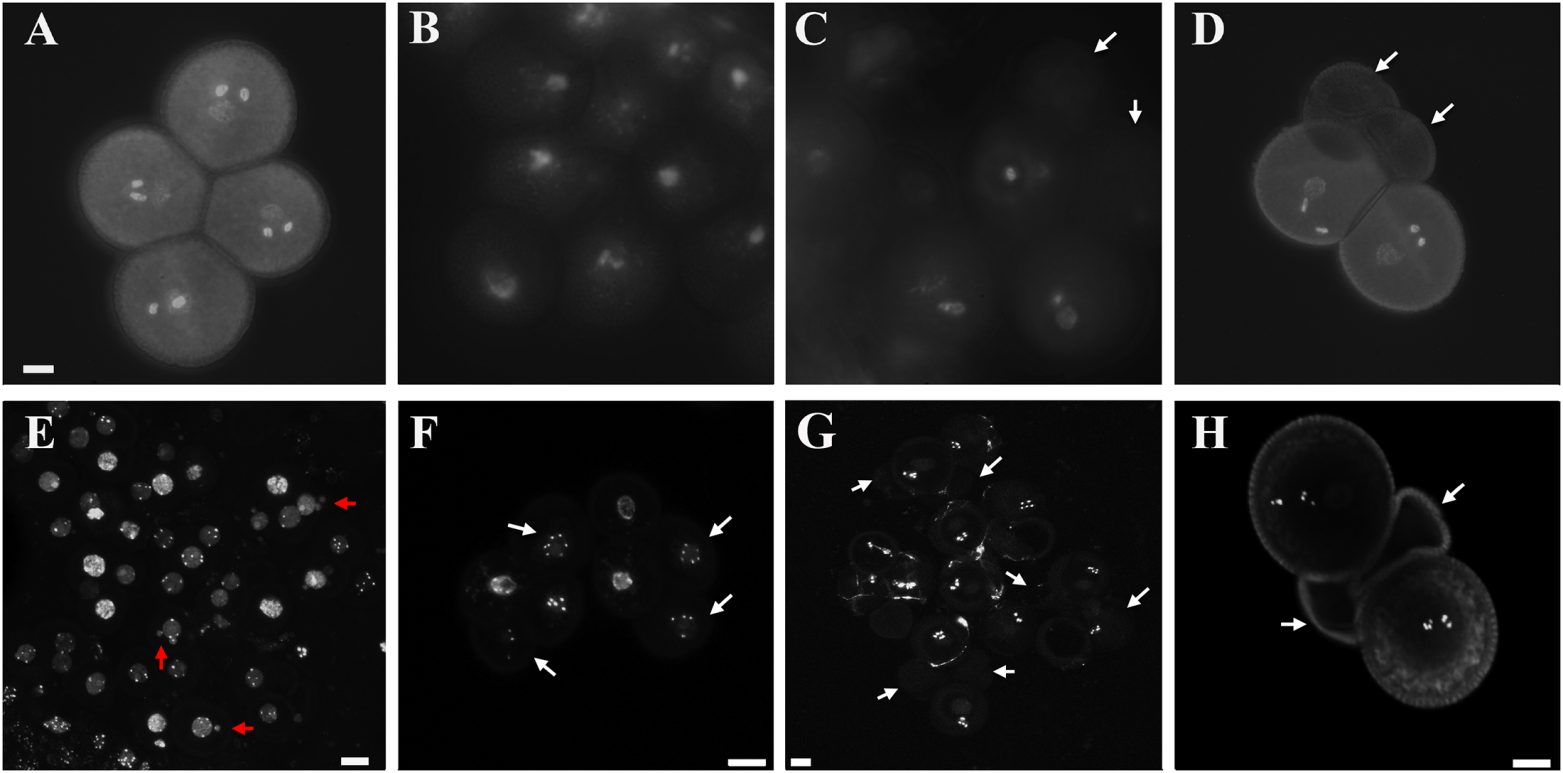
Defects in early pollen development in the *HAP1/hap1-2* mutant. A–D: Pollen stained by DAPI. (A): *qrt* mature pollen grains; (B): *qrt;HAP1/hap1-2* in unicellular stage; (C): *qrt;HAP1/hap1-2* in bicellular stage; and (D): *qrt;HAP1/hap1-2* mature pollen grain. E—H: HTR12GFP showing the distribution of centromere histone H3 in *qrt;HAP1/hap1-2*. Bright dots show detected HTR12GFP representing condensed chromosomes in microspores (E-F), bicellular stage (G) and the mature pollen grain (H) in the *qrt;HAP1/hap1-2* mutant. Recognizable mutant haploids are indicated by white arrow. Examples of micronuclei are indicated by red arrow. Scale bar = 10 μm.

All *qrt* pollen grains exhibited a tricellular pattern as indicated by DAPI staining (Fig 2A). When tetrads from the *HAP1/hap1-2* flower were examined, the nucleus in the *hap1-2* mutant pollen was mostly indistinguishable from the wild type pollen at unicellular stage (Fig 2B). Starting from bicellular stage, the nucleus signal was very faint in the mutant pollen while both nuclei were clearly distinguishable in the wild type pollen (Fig 2C). In the mature pollen, the two mutant pollen grains did not have any detectable DNA and looked shriveled, whereas the two wild type pollen grains showed a perfect tricellular pattern like in *qrt* (Fig 2D). A centromere-specific histone H3, HTR12 [30], exhibited an expected pattern of 5 distinct chromosome dots in the nucleus of each *qrt* microspore (Fig 2E), whereas some of the HTR12 dots in the *HAP1/hap1-2* mutant microspores were excluded from others in the nucleus and formed micronuclei (Fig 2E). Although the fate of these micronuclei was not further studied, any chromosome-level genetic information loss can cause detrimental effects in later steps of pollen development. In some of microspores, there were uneven numbers of chromosomes (Fig 2F) indicating possible mistakes during chromosome segregation during meiosis II. There is no detectable HTR12 in mutant pollen at either bicelluar stage or upon maturation (Fig 2G and 2H). Based on these observations, the molecular difference introduced by *hap1-1* and *hap1-2* explains the reasons for the distinct pollen phenotypes observed in these two mutants.

### AtMagoΔC protein lacks substantial secondary structures at the C-terminus

Since the *AtMagoΔC* transcript is stably present in *HAP1*/*hap1-2* plants, it may be translated into a viable protein, AtMagoΔC. Due to a lack of antibodies specific for the N terminal domain of AtMago (NTD), we were not able to study if AtMagoΔC protein is made and stable. The following work is based on the assumption that AtMagoΔC is a stable protein. The full length AtMago protein contains 150 amino acids folding into 3 α-helixes (A, B and C) and 6 β-pleated sheets (1–6) in the order of β1-β2-β3-β4-αA-β5-β6-αB-αC [13]. AtMagoΔC only has 102 amino acids, with the beginning 72 amino acids identical to that of AtMago and the rest introduced by the retained intron (S1B Fig). Therefore, the αB, αC, β5 and β6 in the C terminal domain (CTD) of AtMago are absent in AtMagoΔC, while the αA helix and β1-β4 sheets in NTD of AtMago are retained in AtMagoΔC. This speculation was confirmed by homology modeling against the protein structure of the *Drosophila* Mago (DmMago) (S1C Fig). This substantial truncation could potentially compromise function of AtMagoΔC if it took the place of AtMago during the EJC assembly.

### Heterodimerization is compromised in AtMagoΔC-AtY14 *in vitro*

**Fig 3:**
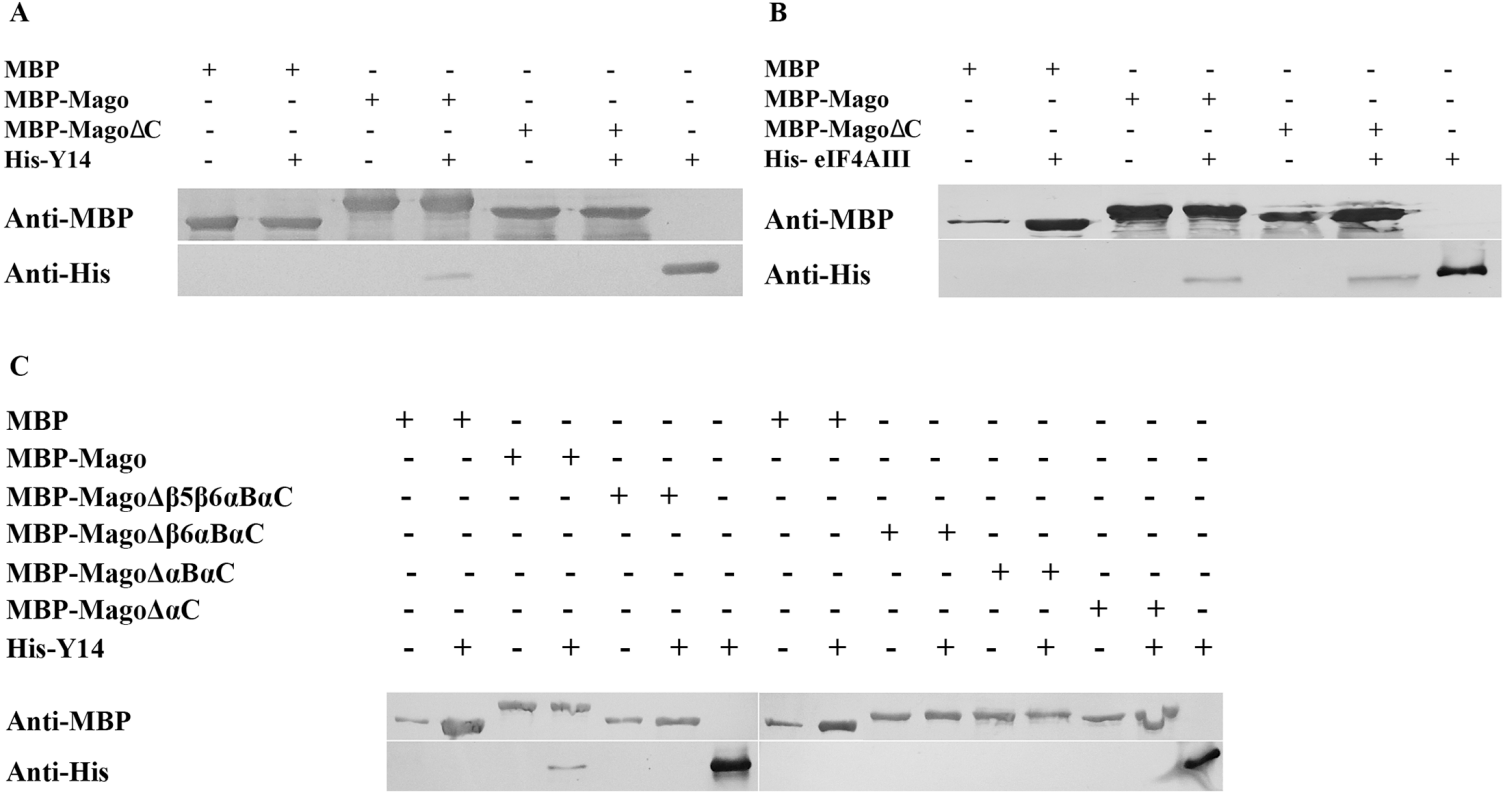
CTD of AtMago is required for AtMago-AtY14 interaction, but not for AtMago-AteIF4AIII. Western blots showing results from *in vitro* pull-down assay for AtMagoΔC-AtY14 (A), AtMagoΔC-AteIF4AIII (B), and various AtMago C terminal truncations-AtY14 (C).

Since Mago and Y14 form a stable heterodimer prior to binding to eIF4AIII and BTZ, the immediate hypothesis is that AtMagoΔC has an impaired function in binding to AtY14. AtMagoΔC and AtY14 were fused with maltose binding protein (MBP) and 6xHis-tag respectively, expressed in *E*. *coli* and purified individually. Results from an *in vitro* pull-down assay showed that MBP was not able to bind to His-AtY14, while MBP-AtMago bound to His-AtY14 readily (Fig 3A). When MBP-AtMagoΔC was incubated with His-AtY14, no detectable His-AtY14 was found in the pull-down product, while MBP-AtMagoΔC was able to bind to AteIF4AIII as MBP-Mago does (Fig 3A and 3B). To investigate this further, the αB, β5 and β6 sequences were added one at a time to AtMagoΔC, and their binding to AtY14 was tested. None of them was able to restore the binding to AtY14 (Fig 3C). This leads to the conclusion that the αC helix in the CTD of Mago is necessary for binding to AtY14. Taken together, C-terminal truncation abolished the formation of AtMago-AtY14 heterodimer, but does not alter the binding between AtMago and AteIF4AIII. This could subsequently affect the integrity of EJC during the assembly.

### Both AtMago and AtMagoΔC were detected in P-bodies

**Fig 4:**
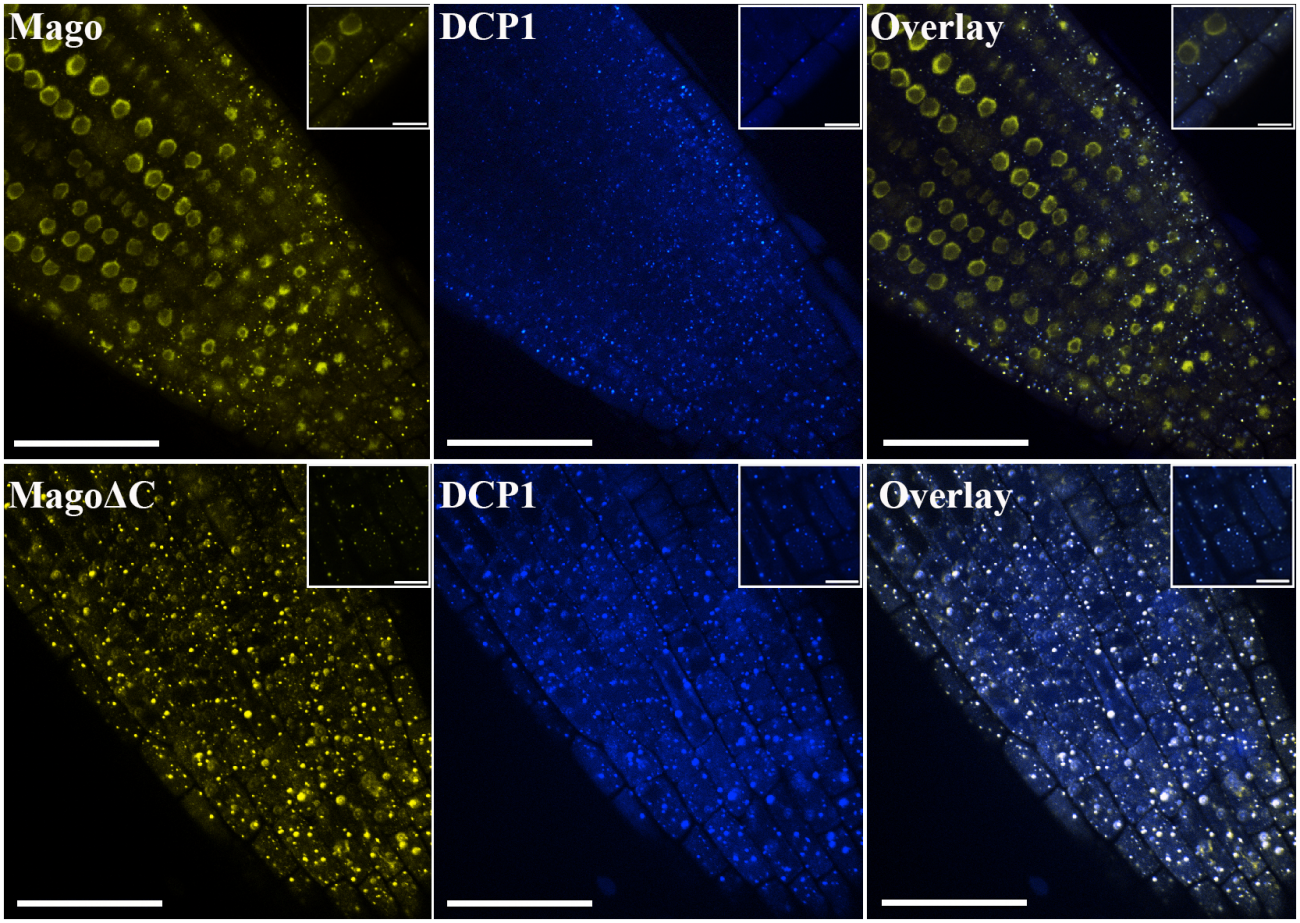
Both AtMago and AtMagoΔC are detected in P-bodies. Scale bar = 50 μm. A zoom-in view is shown for each image with a scale bar of 10 μm.

Mago plays an important role during the EJC assembly. In animal systems, the current model states that after the EJC forms a complex with an mRNA in the nucleus, the complex is exported into cytosol. During translation, proteins within the complex disassociate from the mRNA via a disassociation factor, PYM, and are recycled back to nucleus [31]. AtMago has been found in both the nucleolus and the nucleoplasm [32]. If the existing animal model is applicable to plants, AtMago should be observed in cytoplasm as well. It is also important to find out whether AtMagoΔC is localized in nucleus or the cytoplasm. In stable transgenic lines, GFP-AtMago was detected in both nucleus and numerous unknown cytoplasmic dots. These dots appeared to colocalize with a P-body marker, Decapping 1 (DCP1) (Fig 4) GFP-AtMagoΔC exhibited an identical expression pattern in cytoplasm to that of GFP-AtMago (Fig 4). This suggests that AtMago may be exported from nucleus to cytoplasm, and both AtMago and AtMagoΔC are present in the same cytoplasmic location, P-bodies.

### NMD and EJC synergistically regulate male gametophyte development

**Fig 5:**
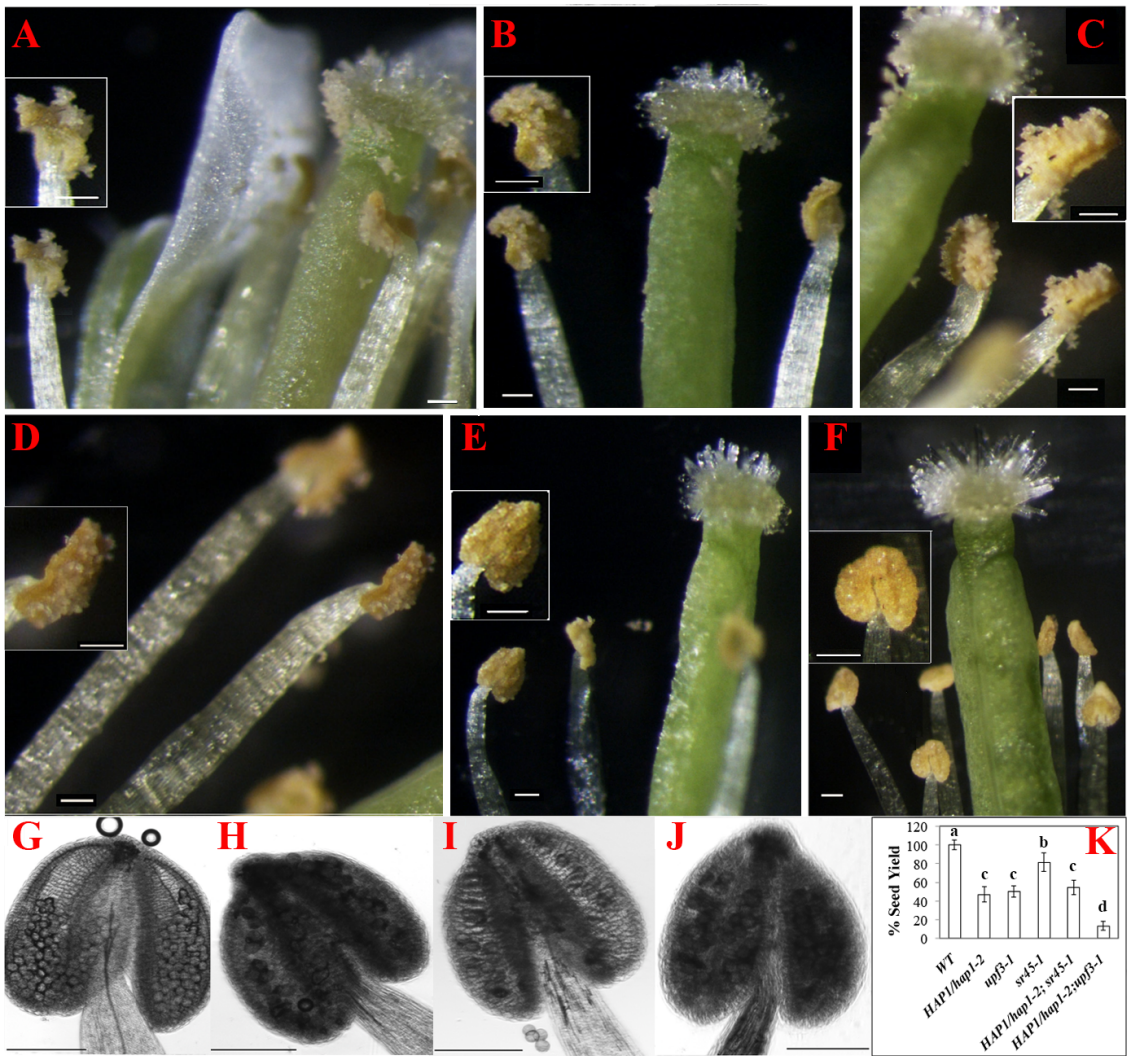
Morphology of flower and anther from different EJC mutants, and their seed yield. Stage 15 flowers from *qrt* (A), *qrt;HAP1/hap1-2* (B), *qrt;upf3-1* (C), *qrt;sr45-1* (D), *qrt;sr45-1; HAP1/hap1-2* (E), *qrt;upf3-1;HAP1/hap1-2* (F) mutant plants. Small inserts show a close view of representative anthers. Pollen formation within the anther from *qrt* (G), *qrt;HAP1/hap1-2* (H), *qrt;upf3-1* (I), *qrt;upf3-1;HAP1/hap1-2* (J). Scale bar = 100 μm. (K) Seed yield as a percentage to WT (*qrt*) was calculated from 16 siliques. Student t-test with Bonferroni correction was used for statistical analysis. Statistical significance (*p*<0.001) is shown as a-d.

The identical cytoplasmic distribution pattern between AtMago and AtMagoΔC leads to the question how the *HAP1/hap1-2* mutant responds to additional defects in either the splicing or the NMD pathway in plants. We crossed the *qrt;HAP1/hap1-2* plant with either the *sr45-1* mutant or with the *upf3-1* mutant. SR45 is orthologous to RNPS1 in *Arabidopsis* [24]. The *sr45-1* mutation increases the abundance of alternative splicing, especially for SR protein gene transcripts, while the *upf3-1* mutation causes the accumulation of NMD-targets, including alternatively spliced SR protein gene transcripts [33]. The *qrt* plant was able to produce and release viable and fertile pollen tetrads (Fig 5A and 5G), and yielded plenty of seeds (Fig 5K). The *qrt;upf3-1* and the *qrt;sr45-1* mutant plants released pollen tetrads comparable to *qrt* (Fig 5C, 5D and 5I). The *qrt;HAP1/hap1-2* plant only produced and released pollen dyads (Fig 5B and 5H). The *qrt;sr45-1;HAP1/hap1-2* plant released pollen dyads similarly to the *qrt;sr45-1* mutant, and produced comparable number of seeds as *qrt;HAP1/hap1-2* plants (Fig 4E). Surprisingly, there was a delay in pollen release from the *qrt;upf3-1;HAP1/hap1-2* anther (Fig 5F). Although the pollen dyads were produced within the anther just like in the *qrt;HAP1/hap1-2* plant (Fig 5J), most anthers showed delayed dehisce in the *qrt;upf3-1;HAP1/hap1-2* plant. The surface of these anthers was smooth and had no signs of rupture for longer time when the carpel was ready to receive pollen (Fig 5F). But they eventual opened, and pollen grains were released. Because of the anther and the carpel were out of synchronicity, the seed yield in the *qrt;upf3-1;HAP1/hap1-2* plant was also dramatically decreased compare to their single mutant parents (Fig 5K). Together, these data showed that a combination of impaired NMD and the lack of CTD in AtMago causes a more severe sterility, which suggests a synergistic relationship between the Mago and NMD functions in Arabidopsis.

### Mutants affect alternative splicing pattern of a subset of SR protein gene transcripts

**Fig 6:**
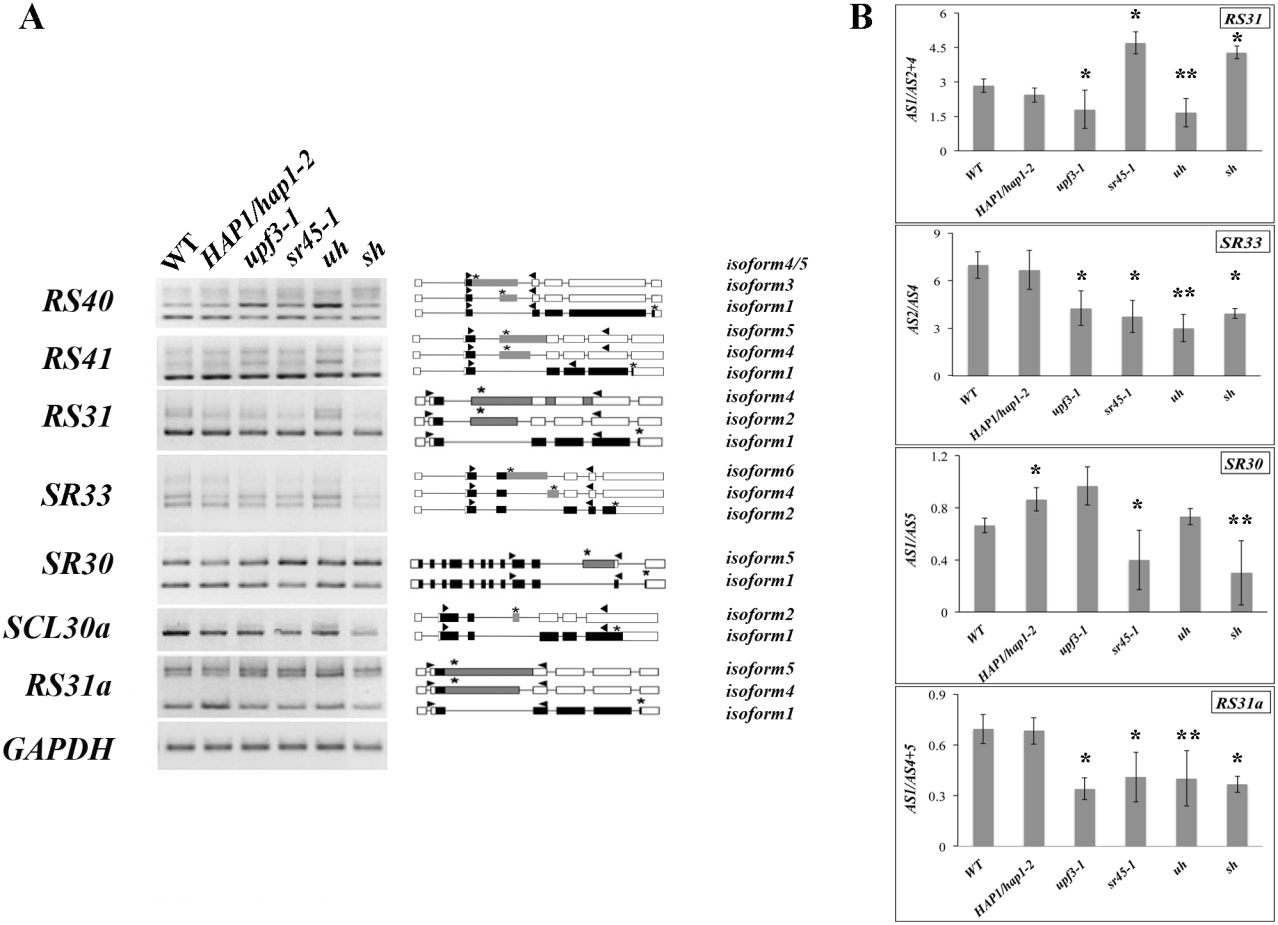
Alternative splicing pattern of SR protein genes in WT, *HAP1/hap1-2*, *upf3-1*, *sr45-1*, *upf3-1;HAP1/hap1-2* (*uh*) and *sr45-1;HAP1/hap1-2* (*sh*) mutant inflorescence tissues. (A) Alternative splicing pattern was examined on *RS40*, *RS41*, *RS31*, *SCL33*, *SR30*, *SCL30a*, *RS31a*. *GAPDH* was used as control. Each band was corresponding to the each splicing isoform. The UTRs were presented by open boxes; the exons were presented by black boxes; the alternatively spliced exons were presented gray boxes, the introns were presented by lines. The position of gene-specific primers was shown by black arrowhead and the stop codons were shown as *. (B) The alternative splicing pattern of selective SR protein gene transcripts was confirmed by qPCR. The ratio of different splicing isoforms (AS) of each genotype was compared to WT (*qrt*) for student t-test (*n* = 3). * represents *p*<0.05 and ** represents *p*<0.01.

As a mechanism to introduce aberrant transcripts as well as diverse viable transcripts, alternative splicing is regulated by splicing factors, such as SR proteins. Splicing factors are involved in the regulation of both constitutive splicing and alternative splicing [34, 35]. In *Arabidopsis*, the transcript of most SR protein genes is also the target of alternative splicing [36]. Both *sr45-1* and *upf3-1* mutants have a higher complexity and diversity of transcript variants due to an elevated level of alternatively spliced transcripts of SR protein genes [24, 33, 37, 38]. This creates a significant accumulation of aberrant transcripts that could potentially affect the fitness of a plant. In this study, the alternative splicing pattern of *RS40*, *RS41*, *RS31*, *SR33*, *SCL30a* and *RS31a* was more dramatically induced in the *upf3;HAP1/hap1-2* mutant inflorescence tissues than all the other genotypes compared (Fig 6A). Some of these patterns were altered slightly in the *upf3-1* and *sr45-1* mutants. The splicing pattern of almost all the compared SR protein gene transcripts, except for *SR30*, in *HAP1/hap1-2* mutant did not differ much from that of the wild type counterpart, *qrt*. Because the *hap1-2* mutation is recessive, one copy of the functional HAP1 seemed to be sufficient for the EJC function in diploid cells. We were not able to design specific primers for qPCR purpose for the genes shown in Fig 6A, but not in Fig 6B. For the genes we were able to examine, a change in the alternative splicing pattern of *RS31*, *SR33*, *SR30* and *RS31a*, was also observed in *sr45-1* and *sr45-1;HAP1/hap1-2* mutants compared to the wild type (Fig 6A and 6B). The alternative splicing profile change in *RS31*, *SR33* and *RS31a* is correlated with the anther defects observed in the *upf3-1;HAP1/hap1-2* mutant. The expression level of *AtMagoΔC*, *AtMago*, *AtUPF3* and *SR45* was verified in all mutants used in this study confirming the correlation between the expression of these EJC component genes and the alternative splicing pattern of the SR protein genes examined above (S2 Fig).

## Discussion

In this study, we have characterized a new mutation, *hap1-2*. Using this mutation, we demonstrated that the CTD, specifically the αC helix, of AtMago is necessary for AtMago-AtY14 heterodimerization during the EJC assembly; hence, the mutational change disturbed the AtY14-binding domain in AtMago. Because lacking the CTD does not interrupt AtMagoΔC from binding to AteIF4AIII, it is likely that AtMagoΔC can take the place of AtMago during the EJC assembly. Since both AtMagoΔC and AtMago exhibit P-body localization, CTD is not required for AtMago to associate with AteIF4AIII, AtBTZ and RNA targets during the NMD process. However, the formation of the AtMago-AtY14 heterodimer is vital for functions of the EJC *in vivo*. Failure in the heterodimerization in plants leads to pollen abortion and the homozygous lethality. When coupling the *HAP1/hap1-2* mutant with an NMD mutant, only a few pollen gains lead to successful fertilization events and seed formation. To our knowledge, this is the first time when a specific domain function is described and confirmed in AtMago protein both *in vitro* and *in vivo*.

Due to the essential roles that the EJC plays in the cell [3], pollen development provides an excellent system to study the EJC at different levels. It is not a surprise that insufficient AtMago protein would cause a deficiency in pollen formation. However, lacking the CTD, AtMagoΔC clearly raises a much stronger effect, the early abortion of the mutant pollen, than what have been reported in other *AtMago* deficient lines in the past [19, 20]. This early decease is likely to be caused by cell division errors, such as irreversible mistakes that occurred during generative cell division, generative cell formation or even as early as microspore formation (Fig 2). In the *hap1-*2 mutant pollen, AtMagoΔC lacks the CTD and fails to form a heterodimer with AtY14. This very likely disrupts the docking of other peripheral proteins, such as UPF3, on the EJC. Studies have shown that human UPF3b binds to Mago-Y14 at conserved residues, Asp66_Mago_ and Glu68_Mago_, located at the very end of the αA helix of Mago and β2 - β3 loop of Y14 [39]. The counterpart of Asp66 and Glu68 are both glutamic acid in AtMago and AtMagoΔC (S3 Fig). However, AtMagoΔC can’t recruit AtY14 to the EJC. Although a UPF3 binding spot on AteIF4AII was also suggested, mutations within β2 - β3 loop of Y14 severely diminished UPF3b’s ability to bind to the EJC core [40]. If this is a similar situation in Arabidopsis based on homology, it is expected that UPF3-mediated NMD would have a difficult time finding its RNA targets in the *hap1-2* mutant. This speculation is further supported by the more severe infertility in the *upf3-1;HAP1/hap1-2* mutant plants (Fig 5K).

In a previous study, GFP-AtMago has been found in nuclear speckles when transiently expressed in onion epidermal cells by biolistic bombardment [41], while other experiments identified GFP-AtMago in all nuclear compartments, the nucleolus, the nucleoplasm and nuclear bodies in Arabidopsis suspension cells [42]. In this study, stable GFP-AtMago and GFP-AtMagoΔC Arabidopsis transgenic lines were generated, individually. GFP-AtMago exhibited strong signals in the nucleus. In addition, both GFP-AtMago and GFP-AtMagoΔC proteins were detectable in P-bodies (Fig 4). It is likely that this contradiction on GFP-AtMago localization was a result of different systems being used. In yeast, EJC-associated aberrant RNAs have been found in P-bodies [43]. Our results suggested a dual localization pattern for Mago and a strong P-body localization for AtMagoΔC. It is not clear whether AtMago shuttles between two locations and whether it moves as free proteins or exists as RNP if it does shuttle between two places. Questions still remain: are AtMagoΔC-associated transcripts natural NMD targets or correct transcripts that are misplaced in P-bodies by AtMagoΔC? Are AtMagoΔC-associated transcripts degraded in P-bodies the same way as AtMago-associated transcripts? Since most AtMagoΔC signal was detected in the cytoplasm, it may not be a noticeable part of nuclear EJC. The increase in alternative splicing variance of cerntain SR protein gene transcripts in the *upf3-1;HAP1/hap1-2* mutant seem to support the scenario that the alternative variants in the mutants are not being degraded as much as in the wild type plants (Fig 6A and 6B).

As a central hub for other peripheral proteins, the EJC is coupled with alterative splicing via RNPS1 and NMD via UPF3 [4]. AtMago is expressed in nucleus (Fig 4). It is known that AtRNPS1 is expressed in all nuclear compartments, and AtUPF3, AtUPF2 and aberrant transcripts are enriched in the nucleolus [32, 44]. Studies in animal cells have shown that the identification of aberrant mRNAs requires the core EJC assembly [45]. It is not clear if this holds true in plant cells. If either splicing regulation or NMD were crippled when EJCs are present in the nucleolus, what would happen to the plant? When increasing alternative splicing events with both AtMago and AtMagoΔC being present as in *sr45-1;HAP1/hap1-2* somatic cells, mRNAs of SR protein genes in the *sr45-1;HAP1/hap1-2* mutant have a similar splicing pattern compared to the *sr45-1* single mutant (Fig 5A and 5B). It has been shown that there is a strong coupling effect between RNPS1 and UPF-triggered NMD [33, 46], and the Mago-Y14 heterodimer provides an anchor for them to bind [4]. Since the *sr45-1;hap1-*2 mutant pollen aborts early, it is impossible to tell whether *sr45-1* induced aberrant transcripts are degraded. We looked at the diploid anther but found no new symptoms. It indicates that without RNPS1, NMD is still able to identify and degrade some aberrant transcripts, especially those affecting anther development. Both AtMago and AtMagoΔC are expressed in *HAP1/hap1-2* diploid cells, the presence of AtMago in the nucleus should lead to correct EJC assembly for NMD to occur. Therefore, the effects of *sr45-1* on alternative splicing is not amplified in the *qrt;sr45-1;HAP1/hap1-2* mutant.

Strikingly, when NMD is impaired while both AtMago and AtMagoΔC being present as in *upf3-1;HAP1/hap1-2* plant, most mutant anthers fail to dehisce early enough. In all the known splicing isoforms produced from SR protein genes, half of them are predicted as potential targets for NMD [36]. In turn, they regulate the splicing of other transcripts in plants. Most of the examined SR protein gene transcripts are affected similarly in both *upf3-1;HAP1/hap1-2* and *upf3-1* mutants (Fig 6A and 6B). This indicates a minor role of Mago in changing the homeostasis of splicing variances for SR protein genes, at least in a heterozygous context. However, the *upf3-1;HAP1/hap1-2* mutant has a much stronger sterility than either of the single mutant. This suggests that Mago may have more contribution in ways other than being a platform for splicing and UPF3-mediated NMD in anther development.

In conclusion, we found that the CTD of AtMago is crucial for binding to AtY14 and the subsequent EJC assembly and function, and Mago works synergistically with UPF3-mediated NMD pathway to regulate anther and male gametophyte development in Arabidopsis. It would be interesting to see how AtMagoΔC affects the docking of other peripheral proteins. And whether it leads to normal NMD in P-bodies. This will shed light on our understanding in how the structural change in the EJC determines its functions as a whole.

## Supporting Information

S1 Fig *AtMagoΔC* sequence and a comparison of AtMago and AtMagoΔC proteins. A. *AtMagoΔC* sequence from 3’ RACE. B. Alignment of the amino acid sequence between AtMago and AtMagoΔC by ClustalW. Identical amino acids found in two sequences are indicated by *. Similar amino acids aligned between two sequences are indicated by: or. Amino acids that compose of β-sheets 1–4 were highlighted in gray and α-helices were highlighted in yellow. C. Homology modeling of the protein structure of AtMago (left) and AtMagoΔC (right).
(TIF)

S2 Fig Expression of *AtMagoΔC*, *AtMago*, *AtUPF3* and *SR45* in *WT*, *HAP1/hap1-2*, *upf3-1*, *sr45-1*, *upf3-1;HAP1/hap1-*2 (*uh*) and *sr45-1;HAP1/hap1-2* (*sh*) mutant inflorescence tissues. *GAPDH* was used as control.
(TIF)

S3 Fig Mago protein sequences from various species and the conserved secondary structure. The secondary structure of Mago protein and the alignment of the ten protein sequences from given species–*Chamydomonas reinhardtii*, *Physcomitrella patens*, *Marsilea vestita*, *Arabidopsis thaliana*, *Oryza sativa*, *Caenorhabditis elegans*, *Drosophila melanogaster*, *Mus musculus* and *Homo sapiens*. The alignment was performed with CLC Sequence Viewer 6. The α-helixes were shown in rods. The β-pleated sheets were shown in block arrows. The amino acid sequence retained in AtMagoΔC was indicated by ☐. The amino acids responsible for the Mago-Y14 interaction are highlighted with *.
(TIF)

S1 Table A list of sequences of gene specific primers. (XLS)
