# Supplementary figures and images for: A New Mutation, hap1-2, Reveals a C Terminal Domain Function in AtMago Protein and Its Biological Effects in Male Gametophyte Development in Arabidopsis thaliana
Source: PLoS One. 2016 Feb 11;11(2):e0148200. doi: 10.1371/journal.pone.0148200 (PMC4750992; doi:10.1371/journal.pone.0148200)

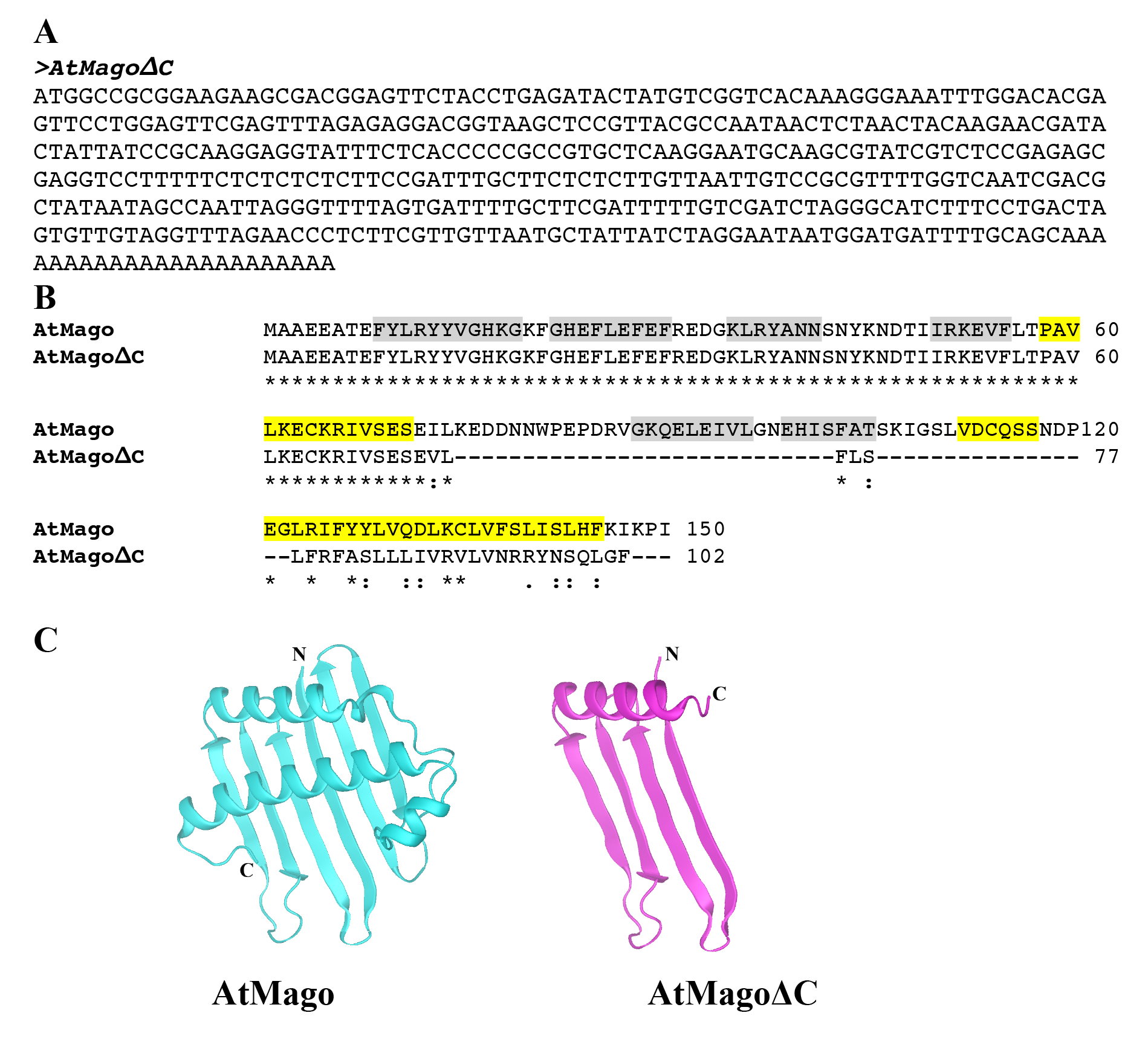

Supplement: S1 Fig — A. AtMagoΔC sequence from 3’ RACE. B. Alignment of the amino acid sequence between AtMago and AtMagoΔC by ClustalW. Identical amino acids found in two sequences are indicated by *. Similar amino acids aligned between two sequences are indicated by: or. Amino acids that compose of β-sheets 1–4 were highlighted in gray and α-helices were highlighted in yellow. C. Homology modeling of the protein structure of AtMago (left) and AtMagoΔC (right). (TIF) [file pone.0148200.s001.tif]

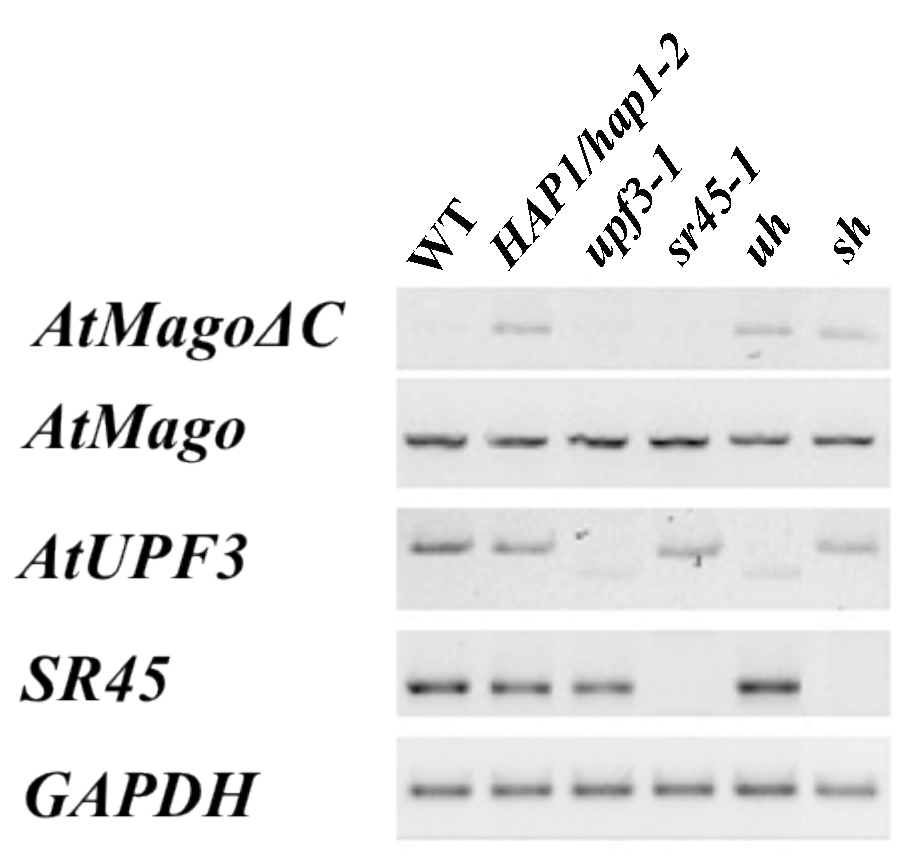

Supplement: S2 Fig — GAPDH was used as control. (TIF) [file pone.0148200.s002.tif]

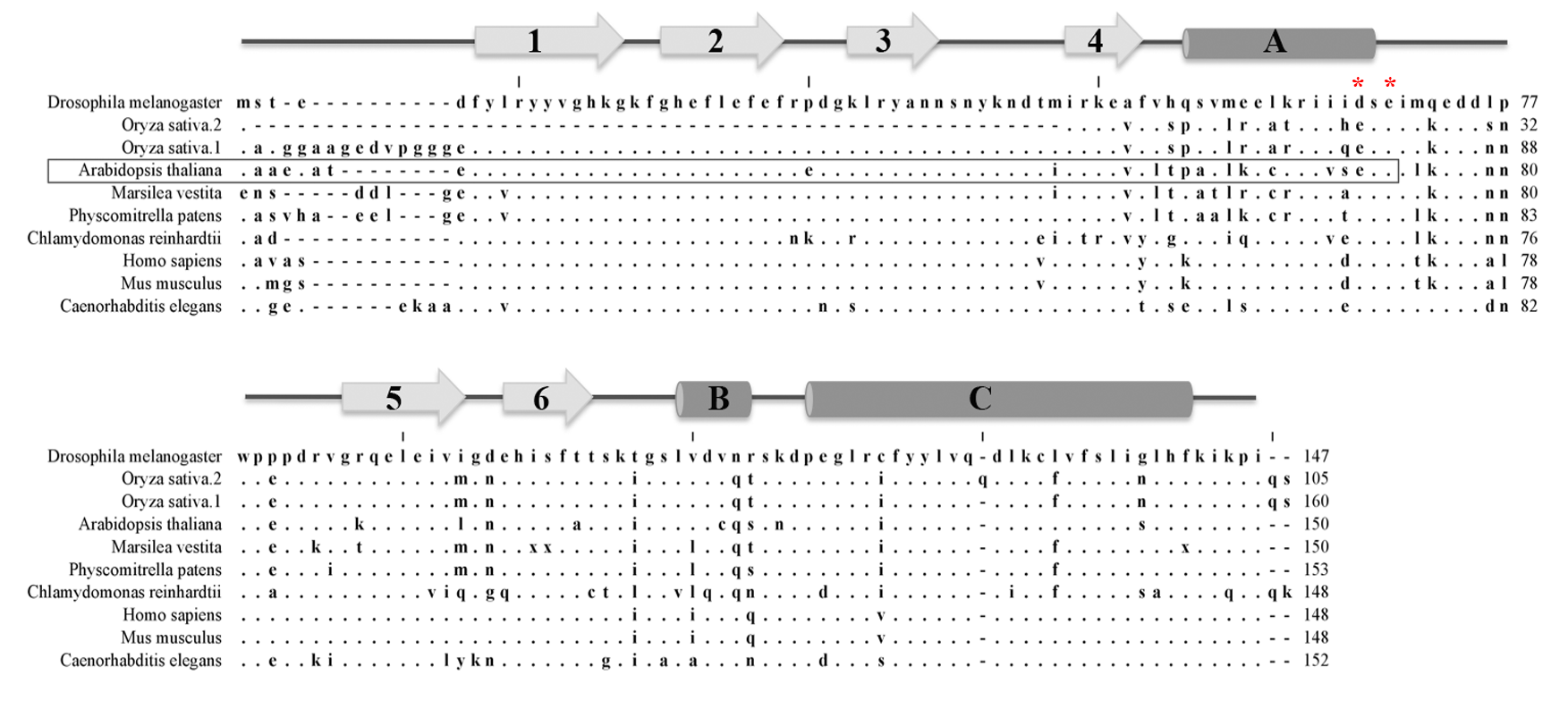

Supplement: S3 Fig — The secondary structure of Mago protein and the alignment of the ten protein sequences from given species–Chamydomonas reinhardtii, Physcomitrella patens, Marsilea vestita, Arabidopsis thaliana, Oryza sativa, Caenorhabditis elegans, Drosophila melanogaster, Mus musculus and Homo sapiens. The alignment was performed with CLC Sequence Viewer 6. The α-helixes were shown in rods. The β-pleated sheets were shown in block arrows. The amino acid sequence retained in AtMagoΔC was indicated by ☐. The amino acids responsible for the Mago-Y14 interaction are highlighted with *. (TIF) [file pone.0148200.s003.tif]
